# The multipartite mitochondrial genome of *Fallopia multiflora* (Caryophyllales: Polygonaceae)

**DOI:** 10.1080/23802359.2018.1437796

**Published:** 2018-02-07

**Authors:** Chang-Kug Kim, Yong-Kab Kim

**Affiliations:** a Genomics Division, National Institute of Agricultural Sciences, Jeonju, Korea;; b School of Electrical Information Communication Engineering, Wonkwang University, Iksan, Korea

**Keywords:** *Fallopia multiflora*, Polygonaceae family, mitochondrial genome

## Abstract

*Fallopia multiflora* is an important Oriental herb belonging to the family Polygonaceae. The *F. multiflora* mitochondrial genome consists of two circular chromosomes that were 200,352- and 112,098-nucleotides long. The mitochondrial genome encodes 57 genes, including 34 protein-coding genes, 20 transfer RNA (tRNA) genes, and three ribosomal RNA (rRNA) genes. When accounting for 3 overlapping genes, 38 genes were found in chromosome I, and 22 in chromosome II. The phylogenetic analysis suggests that *F. multiflora* is closely related to *Beta macrocarpa* and *Silene latifolia*.

*Fallopia multiflora* (Hashuoh in Korean) is an important herb in Korea, Japan, and China (Han et al. 2016). We extracted genomic DNA from fresh leaves collected from the Oriental Medicine Resource Center of Wonkwang University (geographic coordinate: N 35°56′38″, E 126°57′16″). The whole body specimen was registered to the National Agrobiodiversity Center (http://genebank.rda.go.kr/) under the voucher number IT 272664. A total of 4.76 Gbp of data was obtained using the Illumina MiSeq platform (San Diego, CA). DNA libraries were constructed from the Illumina paired-end (PE) library using the Illumina platform.

The mitochondrial genome of *F. multiflora* consists of two circular chromosomes, I (GenBank accession number: MF611850), and II (MF611851). The chromosomes were numbered based on the genome size, and the lengths of the chromosomes were 200,352 and 112,098 nucleotides. *Fallopia multiflora* had two mitochondrial genomes. Multipartite mitochondrial genomes have been reported in some plants, animals, and fungi (Wei et al. 2012).

The mitochondrial genome of *F. multiflora* encoded 57 genes, including 34 protein-coding genes, 20 transfer RNA (tRNA) genes, and three ribosomal RNA (rRNA) genes. Of these 57 genes, 38 were found in chromosome I, and 22 in chromosome II. Three genes overlapped. In addition, we identified 15 open reading frames (ORFs) and splicing variants of the *nad1*, *nad2*, and *nad5* genes. Interestingly, the *nad1* and *nad5* splicing genes were located at exons 1, 2, 3 in chromosome II and exons 4, 5 in chromosome I. Phylogenetic relationships of 15 common *F. multiflora* protein-coding sequences were revealed by comparing data with the genomes of eight other reported species in the Pentapetalae subdivision. The phylogenetic analysis suggested that *F. multiflora* is closely related to the *Beta macrocarpa* and *Silene latifolia* groups (Figure 1).

**Figure 1.**
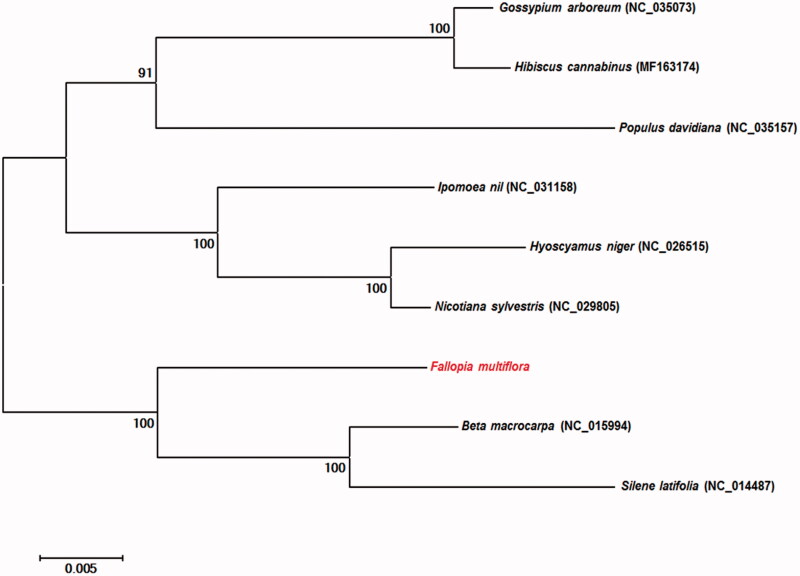
Phylogeny of *F. multiflora* and eight related species based on mitochondrial genome sequences. The phylogenetic tree was constructed using maximum likelihood analysis with 1000 bootstrap replicates based on the full mitochondrial genomes of nine species from the Pentapetalae subdivision.
